# Hierarchical Porous Carbon with Interconnected Ordered Pores from Biowaste for High-Performance Supercapacitor Electrodes

**DOI:** 10.1186/s11671-020-03305-0

**Published:** 2020-04-21

**Authors:** Xiaoxia Bai, Zhe Wang, Jingying Luo, Weiwei Wu, Yanping Liang, Xin Tong, Zhenhuan Zhao

**Affiliations:** 1 Department of Applied Chemistry, Interdisciplinary Research Center of Smart Sensor, School of Advanced Materials and Nanotechnology, Xidian University, Xi’an, 710126 China; 2 Institute of Fundamental and Frontier Sciences, University of Electronic Science and Technology of China, Chengdu, 610054 China

**Keywords:** Hierarchical porous carbon, KIT-6, Biowaste, Supercapacitor

## Abstract

Using biowastes as precursors for the preparation of value-added nanomaterials is critical to the sustainable development of devices. Lignosulphonates are the by-products of pulp and paper-making industries and usually discarded as wastes. In the present study, lignosulphonate is used as the precursor to prepare hierarchical ordered porous carbon with interconnected pores for the electrochemical energy storage application. The unique molecular structure and properties of lignosulphonate ensure the acquisition of high-quality porous carbon with a controllable pore structure and improved physical properties. As a result, the as-prepared hierarchical order porous carbon show excellent energy storage performance when used to assemble the symmetric supercapacitor, which exhibits high-specific capacitance of 289 F g^−1^ at a current density of 0.5 A g^−1^, with the energy density of 40 Wh kg^−1^ at the power density of 900 W kg^−1^. The present study provides a promising strategy for the fabrication of high-performance energy storage devices at low cost.

## Introduction

Supercapacitors hold the promising as energy storage devices for backup systems and various electronics because of their high power density, long cycle life, lightweight in comparison to batteries and conventional capacitors [1–6]. Carbon-based nanomaterials are known for their excellent energy storage performance as active electrode materials in supercapacitors that store electricity through the electrochemical double layer. Their energy storage performance is determined by the physical properties of the electrode active materials, especially the porous structure [7–9]. The macropores (larger than 50 nm) serve as the ion-buffering reservoirs, mesopores (2–50 nm) as the electrolyte ions transport channels and micropores usually as the charge storage sites [10]. Our previous work based on the linear correlation investigation indicates that the specific capacitance and the rate capability are highly related to the volume of both micropores and mesopores [11, 12], respectively. The carbon nanomaterials-based supercapacitors are expected to have a hierarchical porous structure with the balanced distribution of macropores, mesopores, and micropores which are interconnected.

Considerable efforts have been made to prepare hierarchical porous carbon for better energy storage performance [13–16]. Researchers have developed various hard templates including zeolite, MCM-41, MCM-48, SBA-15, SBA-16, and KIT-6 to prepare porous carbon with ordered mesopores [17, 18]. Chemical and physical activation treatments are also widely employed, which usually result in randomly distributed closed pores [13], and hence show poor controllability over the pore structure.

Biomass and biowaste have been chemically and physically activated at elevated temperatures for the preparation of porous carbon [19]. Few of them have been used in the template methods for the synthesis of porous carbon with ordered pore structure [11, 12, 20]. Many porous carbons are prepared from the expensive and non-renewable surfactants and block-copolymers. Lignin is the second most abundant organic materials and the most abundant aromatic polymer existing in plant species [21]. In the paper-making industry, lignin is converted to lignosulphonates during the pulping process and usually discarded as wastes resulting in serious environmental problems [22]. Lignosulphonates are typically small molecules with an aromatic ring and oxygen-containing groups. They typically have much smaller molecular weight than lignin and exhibit excellent water solubility due to the oxygen-containing groups [23]. These merits make lignosulphonates the ideal precursors that can be employed in the template method for the synthesis of value-added porous carbon with ordered pore structure.

In the present study, we used KIT-6 as the hard template for its good interconnectivity of ordered pores and controllability over the pore size to prepare ordered mesoporous carbon combined the post chemical activation to create micropores in the mesoporous structure. Sodium lignosulphonate has been employed as the precursor. The as-prepared hierarchical ordered porous carbon (HOPC) was used to assemble the symmetrical supercapacitor which shows outstanding energy storage performance.

## Methods

### Preparation of KIT-6

The ordered mesoporous silica template (KIT-6) was synthesized according to reference [24]. In a modified procedure, 5.53 g of Pluronic P123 (EO_20_PO_70_EO_20_, MW = 5800, Aldrich) was firstly dissolved in 200 g of deionized water containing 10.9 g of concentrated HCl (35%) in a 250 mL glass bottle. 5.53 g of butanol was then added to the bottle under stirring at 35 ^o^C. After stirring for 1 h, 11.9 g of TEOS (tetraethyl orthosilicate, Aldrich) was added into the above solution, and then the mixture was stirred for 24 h at 35 ^o^C. The bottle was subsequently aged for another 24 h at 100 ^o^C under static conditions. The solid product was collected through filtration and dried at 100 ^o^C without washing. The organic residual was removed by extraction in the mixture of ethanol and HCl, followed by calcination at 550 ^o^C for 6 h.

### Preparation of Ordered Mesoporous Carbon

The ordered mesoporous carbon (OMC) was prepared by using the as-synthesized KIT-6 as the hard template and sodium lignosulphonate as the carbon source. The above-synthesized silica template KIT-6 was used to load lignosulphonate. Typically, 0.6 g of sodium lignosulphonate purchased from Lanyi Reagent (Beijing, China) was dissolved in 15 mL of deionized water, followed by the addition of 0.6 g of KIT-6 template. The mixture was kept stirring for 24 h at room temperature, followed by drying at 70 ^o^C. The dried composite containing silica and sodium lignosulphonate was used as the precursor for carbonization. The carbonization process was conducted at 900 ^o^C for 2 h in Ar with a gas flow rate of 30 sccm. After carbonization, the silica template was removed by immersing the composite into 2.5 M NaOH aqueous solution for 12 h at room temperature. After a washing treatment using a diluted HCl solution and deionized water, mesoporous ordered carbon (abbreviated as OMC) was obtained and recorded as OMC-900 (the digital number refers to the carbonization temperature). OMC-700, OMC-800, and OMC-1000 refer to the obtained samples carbonized at 700 ^o^C, 800 ^o^C, and 1000 ^o^C, respectively.

### Preparation of Hierarchical Ordered Porous Carbon

To prepare the hierarchical ordered porous carbon (abbreviated as HOPC), a post chemical activation process was employed. Briefly, the as-prepared OMC-900 was homogeneously mixed with ZnCl_2_ solution with a weight ratio of carbon to ZnCl_2_ of 1:1 and dried at 110 ^o^C for 6 h. The activation treatment was carried out by heating the composites at 900 ^o^C for 3 h in Ar with a gas flow rate of 30 sccm. To investigate the effect of carbonization temperature, the KIT-6 templates impregnated with sodium lignosulphonate were also carbonized at 700 ^o^C and 800 ^o^C, marked as OMC-700 and OMC-800. For comparison, pure sodium lignosulphonate without any treatment was directly carbonized at the same conditions and the obtained carbon was recorded as lignin-carbon.

### Characterization

The morphology of the as-synthesized KIT-6 template and porous carbon samples were characterized using a Hitachi SU8020 scanning electron microscopy (SEM). The fine porous structure was further examined on a JEOL 2100F transmission electron microscopy (TEM). The low angle XRD diffraction pattern was recorded on a XD-2/XD-3 advance powder X-ray diffractionmeter. The chemical structure was investigated using an ESCALAB250Xi X-ray photoelectron spectroscopy (XPS). Raman characterization was conducted using a HORIBA Science Raman spectroscopy. The Fourier transformed infrared (FTIR) spectra was recorded using a NEXUS 670 FTIR spectroscopy. The porous characteristics of the KIT-6 template and the porous carbon were analyzed by N_2_ adsorption/desorption experiments at 77 K using a Micromeritic ASAP2020 V3.02 H. The specific surface area was measured according to the Brunauer-Emmett-Teller (BET) method, and the pore size distribution was calculated using a slit pore non-local density functional theory (NLDFT) model.

### Electrochemical Measurement

The electrochemical performance of the as-prepared porous carbon samples was examined using a three-electrode configuration. The optimized HOPC was finally investigated using a two-electrode configuration. The working electrode in the three-electrode system was fabricated by physically mixing the as-prepared porous carbon and PVDF which was dissolved in NMP solvent in advance with a weight ratio of carbon to PVDF of 9:1. The homogeneous slurry was cast onto a nickel foil with the coverage area of about 1 cm^2^, followed by drying at 80 ^o^C for 12 h to remove the residual solvent. In the three-electrode testing, a platinum plate (1 cm^2^) and Ag/AgCl were used as the counter electrode and reference electrode, respectively. The working electrode in the two-electrode system was prepared by the same procedure while replacing the nickel foil with nickel foam. Nickel foams with the same loading amount of active electrode materials were used to assemble the symmetrical supercapacitor in which the filter paper was used as the separator. In both the three-electrode and two-electrode configurations, 6 M KOH aqueous solutions were employed as the electrolyte.

Cyclic voltammetry (CV), electrochemical impedance spectroscopy (EIS), and constant galvanostatic charge/discharge were conducted on a Gamry reference 3000 instrument. The CV measurement in the three-electrode configuration was performed at a potential window of − 1 to 0 V versus Ag/AgCl, while the potential window in the symmetric supercapacitor was 0 to 1 V. The EIS characterization was conducted at the AC amplitude of 5 mV in the frequency range from 1 MHz to 0.01 Hz. The specific capacitance from CV curves collected from the three-electrode testing was calculated by the equation *C* = ʃ*I*dt/*mV*. The specific capacitance derived from the CV curve in two-electrode testing and galvanostatic testing was determined via *C* = 4ʃ*I*dt/*MV* and *C* =v4*I*t/*MV*, respectively, where *I* is the discharge current, t is the discharge time, *V* is the working voltage window, *m* is the mass of the active material at the working electrode in the three-electrode configuration, and *M* is the total mass of the active material at the two electrodes in the symmetric supercapacitor. The energy density (*E*) and the power density (*P*) were calculated from galvanostatic charge/discharge testing via *E* = *CV*^2^/2 and *P* = *E*/*t*, respectively, where *C* is the specific capacitance from the two electrodes testing and *t* is the discharge time.

## Results and Discussion

**Fig. 1:**
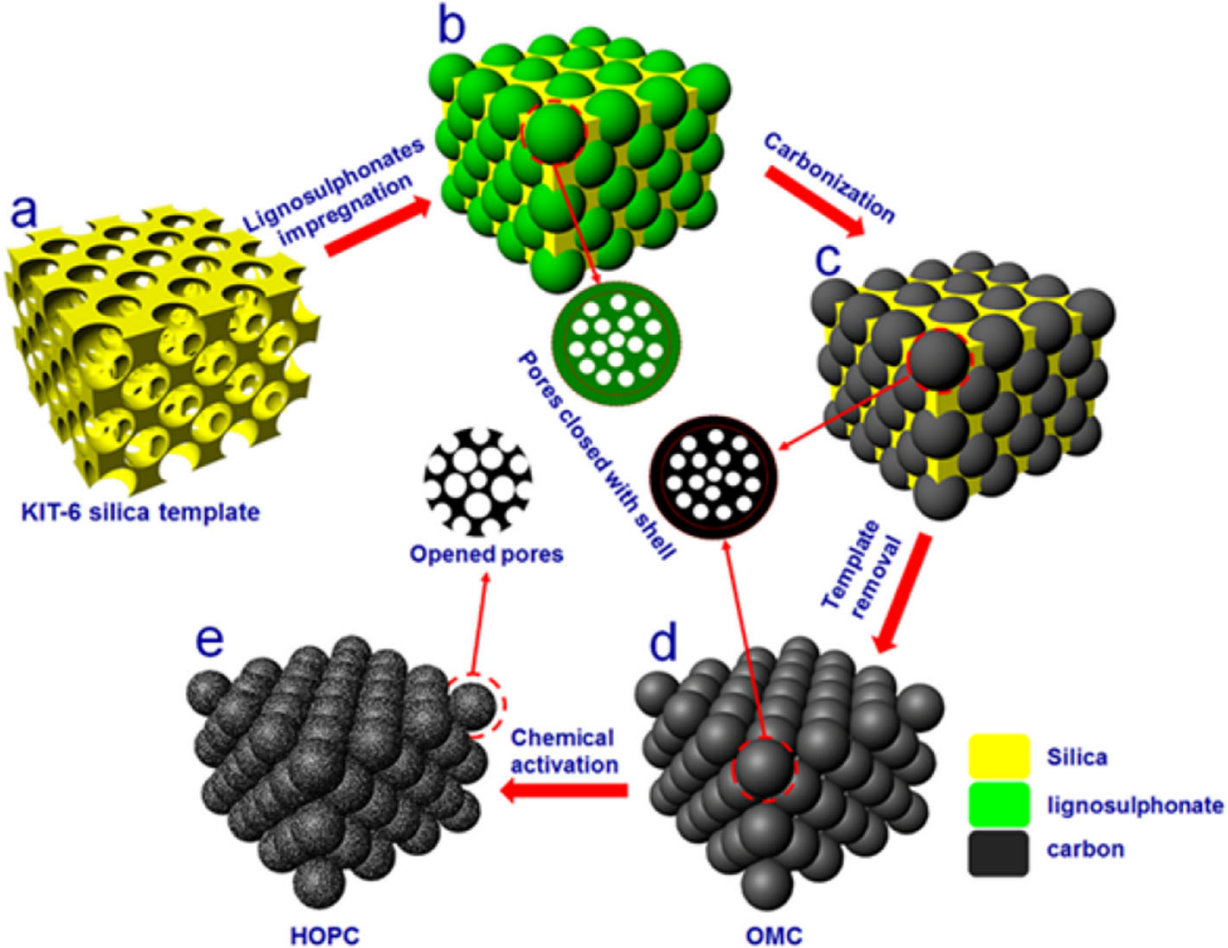
Illustration of the synthetic processes of hierarchical ordered porous carbon (HOPC) using the hard template method combined with post chemical activation

The HOPC sample was synthesized through five steps as illustrated in Fig. 1. (a) The KIT-6 silica template was prepared by a modified method in literature [24]; (b) sodium lignosulphonate was impregnated into the KIT-6 template by immersing the KIT-6 silica template into the sodium lignosulfonate aqueous solution; (c) the KIT-6 loaded with sodium lignosulphonate was carbonized for 2 h in Ar gas. In order to optimize the carbonization process, we performed the carbonization at 700 ^o^C, 800 ^o^C, 900 ^o^C, and 1000 ^o^C. (d) after the carbonization, the KIT-6 silica template was then removed in warm 2.5 M NaOH solution to obtain the ordered mesoporous carbon (OMC); (e) the OMC carbon sample was further chemically activated using ZnCl_2_ at 900 ^o^C for 3 h to prepare HOPC.

**Fig. 2:**
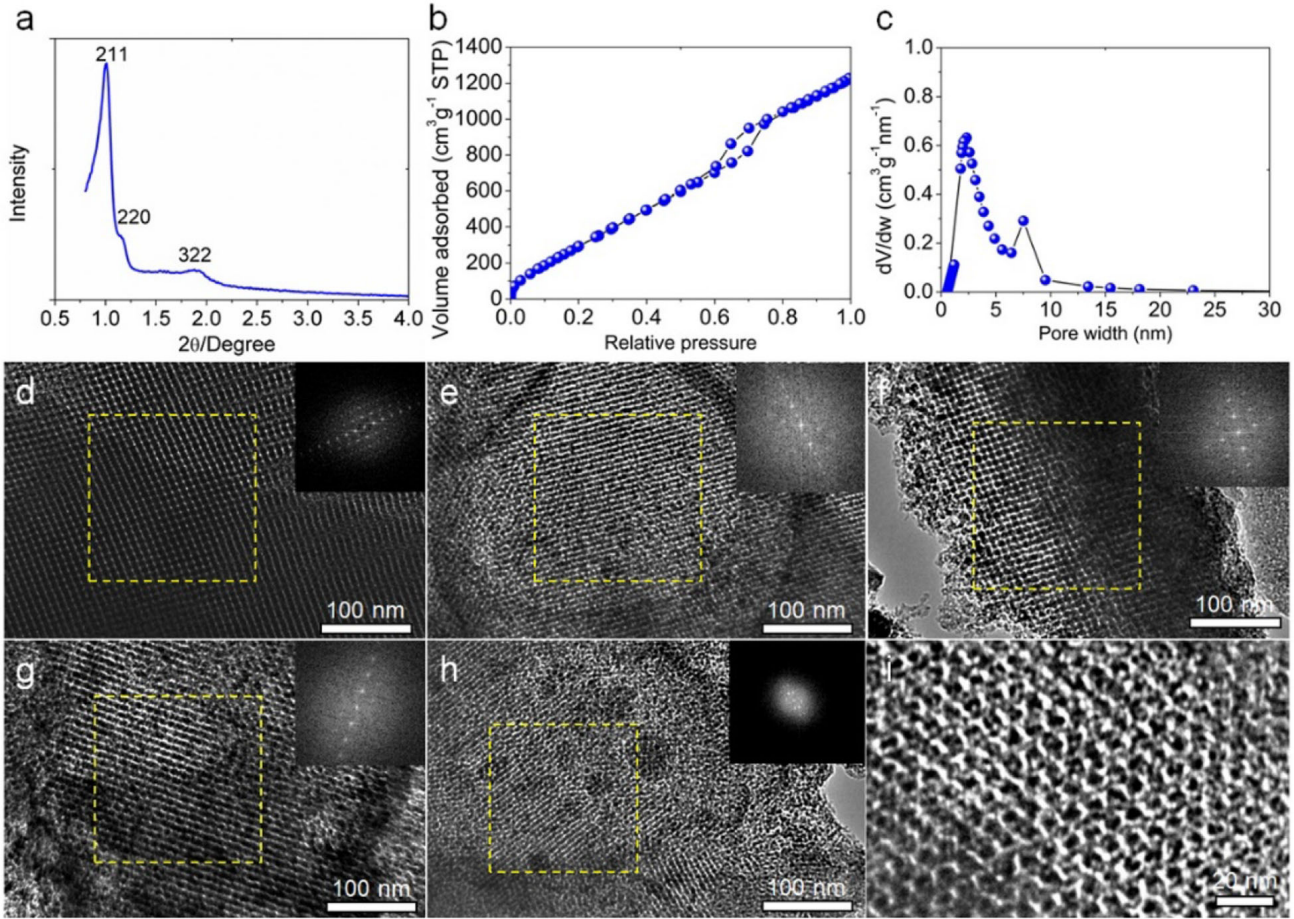
Characterization of the prepared KIT-6 silica template, **a** Low-angle XRD pattern of KIT-6 silica template. **b** N_2_ adsorption/desorption isotherm of KIT-6 template. **c** The corresponding pore size distribution for N_2_ calculated using a slit pore NLDFT model. TEM images of (**d**) KIT-6 silica, the OMC carbon samples prepared at (**e**) 700 ^o^C, (**f**) 800 ^o^C, and (**g**) 900 ^o^C, and the HOPC sample (**h**–**i**). The insert images are the corresponding Fast Fourier Transform patterns of the selected areas

The as-prepared KIT-6 silica template was firstly analyzed using low-angle powder X-ray diffraction pattern (XRD) as shown in Fig. 2a. The as-synthesized KIT-6 silica belongs to cubic *Ia3d* with two characteristic diffraction peaks at 1^o^ and 1.2^o^ of the (211) and (220) planes of the cubic ordered mesoporous structure [24]. The results of the N_2_ adsorption/desorption analysis show that the KIT-6 silica template is consisted of majority mesopores and few micropores, with good interconnectivity (Fig. 2b). The pore size distribution is centered at 2.5 nm and 7.5 nm (Fig. 2c). The BET (Brunauer-Emmett-Teller) surface area is 1481 m^2^ g^−1^ and the total pore volume is 2.62 cm^3^ g^−1^. We further investigated the micromorphology and pore structure of the synthesized KIT-6 template by transmission electron microscopy (TEM) as shown in Fig. 2d. The bright dots represent the pores and the dark area of the walls (silica). The wall thickness and the average pore size are measured to be 3.1 nm and 6.4 nm, respectively. The insert image of the FFT pattern in Fig. 2d confirms the periodically ordered pore structure.

The OMC carbon samples were characterized by SEM and TEM. In Figure S1, the as-prepared OMC carbon samples at different carbonization temperatures display the honeycomb-like morphology with ordered pores. We further used TEM to investigate the microporous structure as shown in Fig. 2e–g. The TEM images show ordered nanodomains. The dark parts are the isolated carbon and the bright parts interconnected pores. The FFT images display sharp and bright spots for all the OMC carbon samples, further confirming the ordered pore structure. The pore size is 2.9 nm, 2.1 nm, and 2.4 nm for OMC-700, OMC-800, and OMC-900, respectively, which is very close to the wall thickness of the silica template. In contrast, the sample prepared from the carbonization of sodium lignosulphonate without the use of the silica template shows no porous structure (Figure S2). It is concluded that ordered mesoporous carbon is successfully prepared by using sodium lignosulphonate and the KIT-6 silica template at the selected carbonization temperatures.

**Fig. 3:**
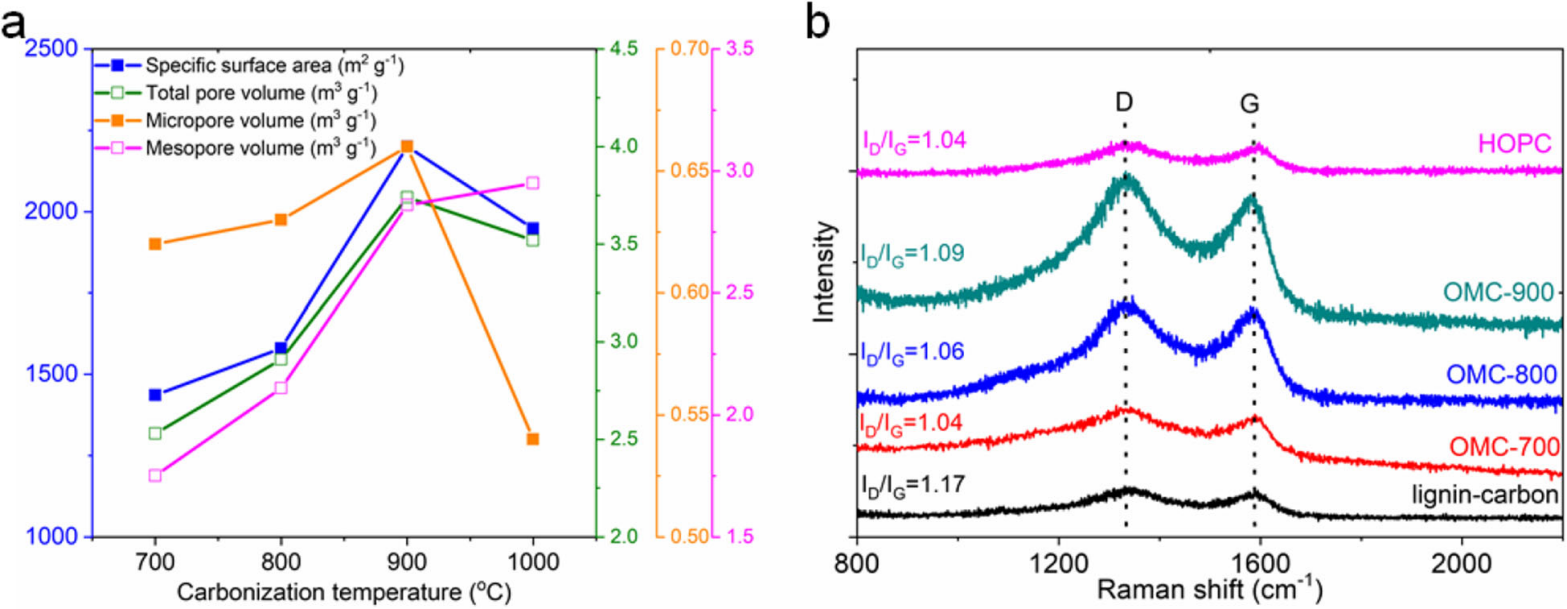
Characterization of the as-prepared OMC carbon and the HOPC samples. **a** The specific surface area, total pore volume, micropore volume, and mesopore volume as the function of carbonization temperature. **b** Raman spectra

**Fig. 4:**
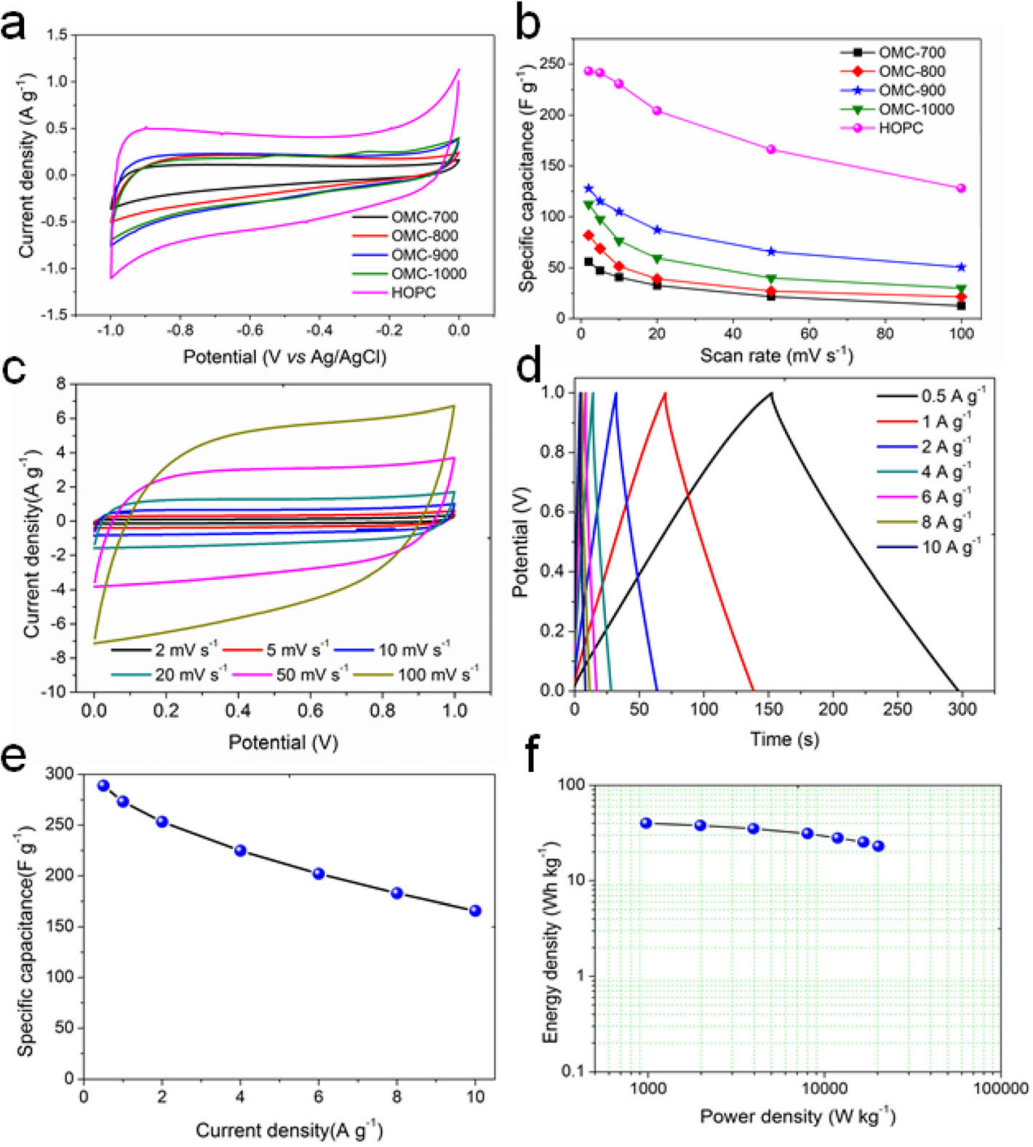
**a** Cyclic voltammetry (CV) profiles of the OMC-700, OMC-800, OMC-900, OMC-1000, and HOPC electrodes at a scan rate of 2 mV s^−1^ using the three-electrode configuration and **b** corresponding specific capacitance. **c** CV profiles of the HOPC electrode in the symmetric two-electrode supercapacitor at scan rates varying from 2 mV s^−1^ to 100 mV s^−1^ in 6 M KOH aqueous electrolyte. **d** Charge-discharge curves at different current density from 0.5 A g^−1^ to 10 A g^−1^. **e** Specific capacitance calculated from the discharge curves from the charge-discharge testing. **f** Ragone plots showing the energy density as a function of power density

Nitrogen adsorption/desorption experiments were conducted to investigate the physical and chemical properties of the as-synthesized OMC carbon samples, as shown in Figure S3. All the isotherm curves have similar intermediate and the adsorbed volume increases at very low relative pressure and rapidly increases with relative pressure, suggesting the coexistence of both micropores and mesopores with the high pore volume for all the as-prepared OMC samples. The pore size distribution for the OMC samples is centered at 0.6 nm and 2.3 nm. We further increased the carbonization temperature to 1000 ^o^C and the isotherm curve of the OMC-1000 sample shown in FigureS4 displays a similar profile, indicating a similar pore structure. Figure 3a shows the specific surface area, total pore volume, micropore volume, and mesopore volume as the function of carbonization temperature. The increase of the specific surface area is related to the increase in the total pore volume. Further increasing the carbonization temperature to 1000 ^o^C, the specific surface area decreases to 1948 m^2^ g^−1^, with decreased micropore volume and increased mesopore volume. It is suggested that the optimal carbonization temperature is 900 ^o^C. Table S1 summarizes the physical properties of the as-prepared OMC carbon samples. It is found that the OMC-900 sample shows the highest specific surface area of 2201 m^2^ g^−1^ and a total pore volume of 3.74 cm^3^ g^−1^.

We therefore conducted chemical activation using ZnCl_2_ as the activation reagent using the OMC-900 sample in order to further create a hierarchical porous structure. As seen from the SEM image in Figure S5, the ordered three-dimensional pore network was partially destroyed after chemical activation to form numerous isolated nanoparticles. The TEM image in Fig. 2e clearly shows ordered pores and the corresponding FFT pattern displays bright spots, indicating the existence of ordered nanodomains. The HRTEM image in Fig. 2f shows that micropores are formed on the walls of mesopores after the chemical activation. The results of N_2_ adsorption/desorption indicate after chemical activation the HOPC sample shows a significantly increased specific surface area of 2602 m^2^ g^−1^, with the micropore volume of 1.03 cm^3^ g^−1^ and mesopores volume of 3.49 cm^3^ g^−1^ (Table S1 and Figure S6a and b).

The properties of lignosulphonates facilitate the formation of ordered mesoporous carbon. Figure S7 shows the representative molecular structure of lignosulphonate, and the hydroxyl groups allow lignosulphonate molecules to occur crosslinking reaction [25]. In the present study, it is proposed that the lignosulphonate molecules are adsorbed onto the hydrophilic silica walls through the hydroxyl groups and crosslinked with each other through hydrogen bonds to form three-dimensional networks. The FTIR spectra of the KIT-6 template, sodium lignosulphonate, and KIT-6 template impregnated lignosulphonates is shown in Figure S8. The peaks of sodium lignosulphonate located at 3429 cm^−1^, 2950 cm^−1^, 1635 cm^−1^, 1514 cm^−1^, 1041 cm^−1^ can be assigned to O–H stretching, C–H stretching, C=O stretching, C–C stretching, and C–O stretching [26], respectively. The peaks of KIT-6 silica template located at 463 cm^−1^, 802 cm^−1^, and 1090 cm^−1^ are assigned to the rocking of Si–O–Si. The peak located at 967 cm^−1^ is due to the vibration of Si–O of the surface silanols [27]. In the spectra of lignosulphonate-silica, the characteristic peaks of lignosulphonate and silica are coexisted, confirming the impregnation of lignosulphonate in silica template. In addition, the peak located at 3429 cm^−1^ in lignosulphonate-silica is broadened, indicating the formation of hydrogen bonding among lignosulphonate molecules through a crosslinking reaction. The adsorbed and oriented lignosulphonate molecules are converted to solid carbon layers during the carbonization process which may induce numerous closed pores, as illuminated in step b and c in Fig. 1. The chemical activation helps to open these closed pores to further improve the physical properties.

Raman characterization was used to investigate the graphite degree of the as-prepared carbon samples and the results are shown in Fig. 3b. All the Raman spectra display characteristic D band and G band located at 1340 cm^−1^ and 1590 cm^−1^, respectively. The intensity ratio of the D band (the disordered carbon) and the G band (ordered carbon) for the OMC samples and the HOPC sample is around 1.06, lower than 1.17 of the carbon sample prepared by the carbonization of lignosulphonate without the use of silica template. This result indicates that the OMC carbon samples have a higher graphite degree than the carbon sample from the direct carbonization of lignosulphonate. The reason is probably ascribed to the above-mentioned crosslinking reaction among the lignosulphonate molecules which helps the orientation of the aromatic rings. The result of the XPS analysis in Figure S9 shows that all the samples mainly contain carbon and oxygen. There is no detectable signal of impurity in the final carbon products. The decomposition of sodium lignosulphonate may give rise to the formation of CO_2_ and Na_2_CO_3_ [28], which can be completely removed during the subsequent washing treatment with deionized water. The C1s core-level can be deconvoluted into four components for all the as-prepared carbon samples. That is, C–O (286.7 eV), C=O (288.0 eV) [29], the sp^2^ carbon (284.8 eV, ordered carbon), and the sp^3^ carbon (285.4 eV, disordered carbon) [30].

The electrochemical performance of the as-prepared OMC carbon samples and the activated HOPC sample were evaluated through a three-electrode configuration in 6 M KOH aqueous electrolyte. The carbon sample from the direct carbonization of lignosulphonate shows negligible energy storage performance (Figure S10). The OMC carbon samples display rectangular CV profiles indicating the improved electrochemical performance (Figure S11 and Fig. 4a). At 2 mV s^−1^, the specific capacitance is 59 F g^−1^, 93 F g^−1^, 130 F g^−1^, and 120 F g^−1^ for OMC-700, OMC-800, OMC-900, and OMC-1000 (Fig. 4b). The OMC-900 electrode shows the best electrochemical performance among all the OMC carbon samples. After chemical activation, the specific capacitance further increases to 243 F g^−1^ for the HOPC sample, almost twice higher than that of OMC-900 sample before activation. The specific capacitance of HOPC in this study is much higher than that of fungi derived carbon (196 F g^−1^ at 5 mV s^−1^) [31], and also higher than that of graphene aerogel based mesoporous carbon prepared from a hard silica template (226 F g^−1^ at 1 mV s^−1^), which decreases to 83 F g^−1^ at 100 mV s^−1^ [32]. For the fungi-derived carbon, the specific capacitance decreases to 90 F g^−1^ at 100 mV s^−1^. However, the specific capacitance of HOPC is still as high as 128 F g^−1^ at the same scan rate.

Impedance measurements were conducted to investigate the conductivity of the samples. Figure S12 reveals the Nyquist spectra of impedance in the frequency range of 1 MHz–0.01 Hz, and the corresponding equivalent circuit which is consisted of the equivalent series resistance (*R*_*s*_), charge transfer resistance (*R*_*ct*_), and the electrochemical double-layer capacitance. The equivalent series resistance *R*_s_ is 0.7 Ω cm^−2^ for all the electrodes, indicating the high electrical conductivity of the samples and high quality of the electrodes. The Nyquist plots indicate that the HOPC electrode shows the lowest charge transfer resistance of 5 Ω.

The deliverable energy and power density is highly related to the frequency-dependent capacitance [33, 34], which can be expressed as follows

$$ C=\frac{1}{jwZ}=\frac{-{Z}_{\mathrm{image}}}{W{\left|Z\right|}^2}-j\frac{-{Z}_{\mathrm{real}}}{W{\left|Z\right|}^2}={C}_{\mathrm{real}}-j{C}_{\mathrm{image}} $$

where *C* and *Z* are the capacitance and resistance, respectively. *Z*_real_ and *Z*_image_ refer to the real and imaginary parts of *Z*. *C*_real_ is the real part of capacitance representing the deliverable capacitance of the electrode materials, and *C*_image_ is the imaginary capacitance related to the irreversible resistivity loss in the device. Figure S13a shows the HOPC sample had the fastest response. The frequency *ƒ* is the character frequency at which *C*_image_ reaches the maximum, and *t = 1/ƒ* is the time constant of the supercapacitor. Both *f* and *t* are the characteristic rate capability. High power density supercapacitors usually have high character frequency *ƒ* and small *t*. Figure S13b shows the plots of *C*_image_ as a function of frequency. The character frequency of the OMC-700, OMC-800, OMC-900, OMC-1000, and HOPC sample is 0.01 Hz, 0.1 Hz, 0.01 Hz, 0.01 Hz, and 0.5 Hz, and the corresponding time constant is 100 s, 10 s, 100 s, 100 s, and 2 s. The HOPC sample shows the highest character frequency and the lowest time constant, indicating the fastest response to power output.

It has been demonstrated that HOPC displays high electrochemical performance. However, for practical applications, three-electrode configuration testing cannot reveal the actual energy storage ability [33, 35]. Therefore, we used HOPC sample to prepare electrodes to assemble symmetric supercapacitors. Figure 4c illustrates the CV curves of the HOPC electrode. The CV profiles exhibit nearly rectangular-shape with good symmetry at all scan rates from 2 mV s^−1^ to 100 mV s^−1^, indicating the good electrochemical properties and rate stability of the HOPC electrode. The galvanostatic charge-discharge measurements at constant current densities were also performed on the as-assembled symmetric supercapacitor and the results are shown in Fig. 4d. The linear voltage versus time profiles during the charge and discharge process show an ideal triangle shape with good symmetry, representing the outstanding energy storage ability of HOPC electrode. When the current density was 0.5 A g^−1^, the discharge time is as long as 150 s, which is corresponding to the specific capacitance of about 289 F g^−1^. The specific capacitance of HOPC from lignosulphonate at 10 A g^−1^ is still as high as 166 F g^−1^, as shown in Fig. 4e. The performance of the HOPC in this study is better than the values of carbon samples in literatures [3, 36–39].

The high-specific capacitance of HOPC is contributed to the low ohmic resistance and charge transfer resistance, which is about 0.6 Ω cm^−1^ and 2.4 Ω cm^−1^, respectively, as shown in Figure S14. The phase angle of the symmetric supercapacitor at the lowest frequency of 0.01 Hz is about 81.7^o^ (Figure S15), which is very close to the value of the ideal supercapacitor (90^o^). The character frequency is about 0.2 Hz, corresponding to the time constant of 5 s. This means the HOPC electrode has a good power delivery ability. The deliverable capacitance is as high as 290 mF (Figure S16). Figure 4f illustrates the Ragone plot of the symmetric supercapacitor. The energy density is in the range of 40 Wh kg^−1^ to 23 Wh kg^−1^ with the power density of about 0.9 kW kg^−1^ to 20 kW kg^−1^.

The high-specific capacitance and energy density of HOPC can be contributed to the optimized pore structure. The HOPC sample contains high pore volume of both the micropores and mesopores. As demonstrated in the previous literatures by correlation analysis [40, 41], the micropores are highly related to the energy storage and the mesopores are highly related to the capacitance retention since the mesopores are mainly responsible for ion transportation. It is also concluded that the mesopores have contributions to the charge storage. Keep the above instruction in mind, we dedicately employed the hard template method and chemical activation to prepare the HOPC sample. The present results not only further demonstrate the above conclusions, but also shows the design for the preparation of high-performance energy storage materials. In order to measure cycleability, the symmetric supercapacitor of using HOPC as the electrode material is repeatedly charged and discharged at 2 A g^−1^ for 3000 cycles (Figure S17). It is found that after 3000 cycles, the specific capacitance is 218 F g^−1^ with a slight decrease from initial 253 F g^−1^ with capacitance retention of 86.2%. The specific capacitance of our HOPC sample from the two-electrode system is higher than and comparable after 3000 cycles with the reported values in Table S2. Therefore, the present study provides a potential route for the development of high-performance supercapacitor electrode-active materials from industrial wastes.

## Conclusion

In this study, we have successfully prepared ordered mesoporous carbon materials using biowaste lignosulphonate as the carbon source using the mold casting technique based on KIT-6 template. During the mold casting process, lignosulphonate can easily be cast into the pores of KIT-6. The crosslinking reaction of lignosulphonate molecules not only increases the pore volume, but also bridges the aromatic rings to promote the graphitization. The as-synthesized ordered mesoporous carbons exhibit high electrical conductivity, high-specific surface area, and pore volume, which are highly dependent on the carbonization temperature. The results of Raman analysis and N_2_ adsorption/desorption experiments show that the OMC-900 sample has the best physical properties. The pore structure of OMC-900 was further optimized through ZnCl_2_ chemical activation to prepare HOPC. The specific capacitance of HOPC in the symmetric supercapacitor was about 289 F g^−1^ with the energy density as high as 40 Wh kg^−1^. The present study indicates lignosulphonate is very suitable to prepare hierarchical ordered porous carbon at low cost with high-performance supercapacitors.

## Supplementary information

Additional file 1: **Figure S1.** Typical SEM images of the as-prepared OMC carbon samples at (a) 700 ^o^C, (b) 800 ^o^C, (c) 900 ^o^C. **Figure S2.** Typical TEM image of the as-prepared carbon sample from the direct carbonization of lignosulphonate without the use of silica template. **Figure S3.** (a) The isotherm curves of nitrogen adsorption/desorption and (b) pore size distribution of the prepared OMC carbon samples at 700 ^o^C, 800 ^o^C, 900 ^o^C. **Figure S4.** (a) The isotherm curves of nitrogen adsorption/desorption and (b) pore size distribution of the prepared OMC carbon sample at 1000 ^o^C. **Figure S5.** SEM image of the HOPC carbon sample prepared from the chemical activation using OMC-900. **Figure S6.** (a) The isotherm curves of nitrogen adsorption/desorption and (b) pore size distribution of the HOPC carbon sample. **Figure S7.** Representative molecular structure of sodium lignosulphonate. **Figure S8.** The FTIR spectra of the as-prepared KIT-6 silica template, the sodium lignosulphonate and the KIT-6 template loaded with lignosulphonates (Lig-silica). **Figure S9.** XPS characterization of the as-prepared carbon samples. (a) the survey scan curves, and (b) the C1s spectra of the lignin-carbon from the carbonization of lignosulphonate without the use of template, and the C1s spectra of (c) OMC-700, (d) OMC-800, (e) OMC-900 and (d) the HOPC sample. **Figure S10.** CV profile and the specific capacitance of the carbon sample prepared by the direct carbonization of lignosulphonate without the use of silica template. **Figure S11.** CV profile of the OMC carbon samples (a) OMC-700, (b) OMC-800, (c) OMC-900, (d) OMC-1000, and the HOPC sample. **Figure S12.** The Nyquist plots of impedance in the frequency range of 1 MHz – 0.01 Hz. The insert refers to the corresponding equivalent circuit. The equivalent circuit contains an ohmic resistance R_s_, which includes the contact resistance of leads, the bulk solution resistance and the sheet resistance of the carbon film, a charge transfer capacitance C_ct_ which is in parallel with the charge transfer resistance R_ct_, and a Warburg diffusion element (W) attributed to the diffusion of electrolyte ions, and the electrical double layer capacitance C_dl_ at the low frequency region. **Figure S13.** (a) The real part of the capacitance C_real_ as a function of frequency and (b) the imaginary part of the capacitance C_image_ as a function of the frequency of the OMC carbon sample and the HOPC sample. **Figure S14.** Nyquist plots of the HOPC symmetric supercapacitor. The insert refers to the magnified Nyquist plots to clearly show the ohmic resistance and charge transfer resistance at high frequency and medium frequency region, respectively. Figure S15 The phase angle as the function of frequency of the HOPC symmetric supercapacitor. **Figure S16.** C_real_ and C_image_ as the function of frequency of the HOPC symmetric supercapacitor. **Figure S17.** Specific capacitance as a function of cycle numbers showing the capacitance retention of the HOPC symmetric supercapacitor. **Table S1.** The specific surface area and pore volume of as-synthesized OMC-700, OMC-800, OMC-900 and HOPC. **Table S2.** Comparison of the specific capacitances of synthesized HOPC electroactive material with recently reported carbonaceous materials. (DOCX 2485 kb)

## Data Availability

All data generated or analyzed in this study are included in the manuscript and the supplementary information files. All the materials are available from the corresponding author on request.

## Abbreviations

HOPC: Hierarchical ordered porous carbon
KIT-6: The ordered mesoporous silica template
OMC: The ordered mesoporous carbon
NMP: N-methyl pyrrolidone
PVDF: Polyvinylidene fluoride
CV: Cyclic voltammetry
EIS: Electrochemical impedance spectroscopy
AC: Alternating current
