# Supplementary material for: Hierarchical Porous Carbon with Interconnected Ordered Pores from Biowaste for High-Performance Supercapacitor Electrodes
Source: Nanoscale Res Lett. 2020 Apr 21;15:88. doi: 10.1186/s11671-020-03305-0 (PMC7174449; doi:10.1186/s11671-020-03305-0)
Supplement: Supplementary file 1 — Figure S1. Typical SEM images of the as-prepared OMC carbon samples at (a) 700 oC, (b) 800 oC, (c) 900 oC. Figure S2. Typical TEM image of the as-prepared carbon sample from the direct carbonization of lignosulphonate without the use of silica template. Figure S3. (a) The isotherm curves of nitrogen adsorption/desorption and (b) pore size distribution of the prepared OMC carbon samples at 700 oC, 800 oC, 900 oC. Figure S4. (a) The isotherm curves of nitrogen adsorption/desorption and (b) pore size distribution of the prepared OMC carbon sample at 1000 oC. Figure S5. SEM image of the HOPC carbon sample prepared from the chemical activation using OMC-900. Figure S6. (a) The isotherm curves of nitrogen adsorption/desorption and (b) pore size distribution of the HOPC carbon sample. Figure S7. Representative molecular structure of sodium lignosulphonate. Figure S8. The FTIR spectra of the as-prepared KIT-6 silica template, the sodium lignosulphonate and the KIT-6 template loaded with lignosulphonates (Lig-silica). Figure S9. XPS characterization of the as-prepared carbon samples. (a) the survey scan curves, and (b) the C1s spectra of the lignin-carbon from the carbonization of lignosulphonate without the use of template, and the C1s spectra of (c) OMC-700, (d) OMC-800, (e) OMC-900 and (d) the HOPC sample. Figure S10. CV profile and the specific capacitance of the carbon sample prepared by the direct carbonization of lignosulphonate without the use of silica template. Figure S11. CV profile of the OMC carbon samples (a) OMC-700, (b) OMC-800, (c) OMC-900, (d) OMC-1000, and the HOPC sample. Figure S12. The Nyquist plots of impedance in the frequency range of 1 MHz – 0.01 Hz. The insert refers to the corresponding equivalent circuit. The equivalent circuit contains an ohmic resistance Rs, which includes the contact resistance of leads, the bulk solution resistance and the sheet resistance of the carbon film, a charge transfer capacitance Cct which is in parallel with the cha [file 11671_2020_3305_MOESM1_ESM.docx]

Hierarchical Porous Carbon with Interconnected Ordered Pores from Biowaste for High Performance Supercapacitor Electrodes

Xiaoxia Bai,^a^ Zhe Wang,^a^ Jingying Luo,^a^ Weiwei Wu,^a^ Yanping Liang,^a^ Xin Tong,^*b^ Zhenhuan Zhao^*a^

^a^ Department of Applied Chemistry, Interdisciplinary Research Center of Smart Sensor, School of Advanced Materials and Nanotechnology, Xidian University, Xi’an 710126, China.

^b^ Institute of Fundamental and Frontier Sciences, University of Electronic Science and Technology of China, Chengdu 610054, China.

Corresponding author: xin.tong@uestc.edu.cn, zhzhao@xidian.edu.cn


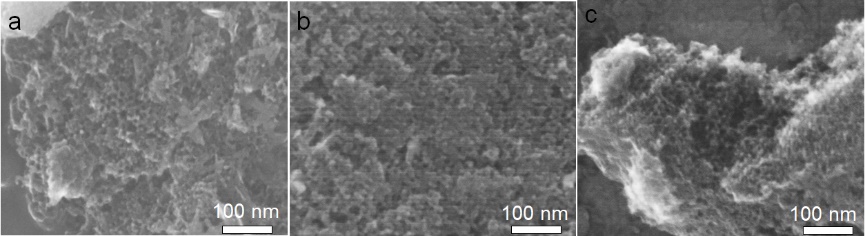


**Figure S1** Typical SEM images of the as-prepared OMC carbon samples at (a) 700 ^o^C, (b) 800 ^o^C, (c) 900 ^o^C.





**Figure S2** Typical TEM image of the as-prepared carbon sample from the direct carbonization of lignosulphonate without the use of silica template.


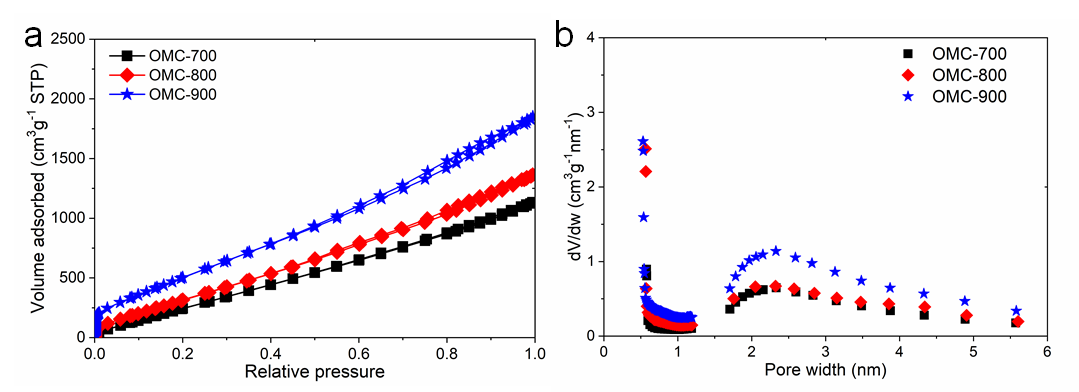


**Figure S3** (a) The isotherm curves of nitrogen adsorption/desorption and (b) pore size distribution of the prepared OMC carbon samples at 700 ^o^C, 800 ^o^C, 900 ^o^C.


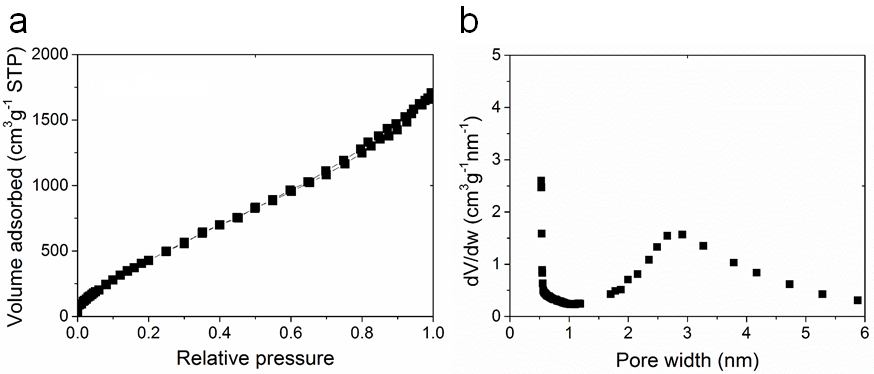


**Figure S4** (a) The isotherm curves of nitrogen adsorption/desorption and (b) pore size distribution of the prepared OMC carbon sample at 1000 ^o^C.

**Table S1** The specific surface area and pore volume of as-synthesized OMC-700, OMC-800, OMC-900 and HOPC.

| **Sample ID** | **Specific surface area**  **(m^2^ g^-1^)** | **Total pore volume**  **(cm^3^ g^-1^)** | **Micropore volume**  **(cm^3^ g^-1^)** | **Mesopore volume**  **(cm^3^ g^-1^)** |
| --- | --- | --- | --- | --- |
| OMC-700 | 1436 | 2.53 | 0.62 | 1.75 |
| OMC-800 | 1580 | 2.91 | 0.63 | 2.11 |
| OMC-900 | 2201 | 3.74 | 0.66 | 2.86 |
| OMC-1000 | 1948 | 3.52 | 0.54 | 2.95 |
| HOPC | 2602 | 4.81 | 1.03 | 3.49 |


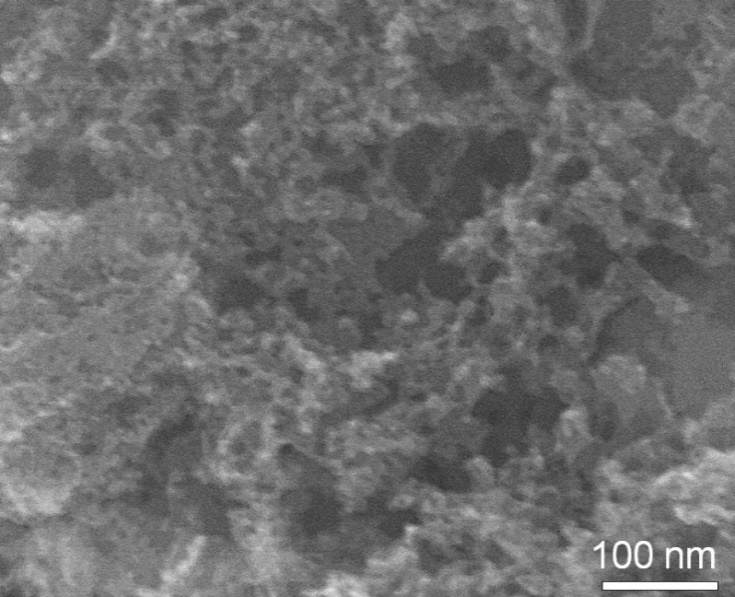


**Figure S5** SEM image of the HOPC carbon sample prepared from the chemical activation using OMC-900.


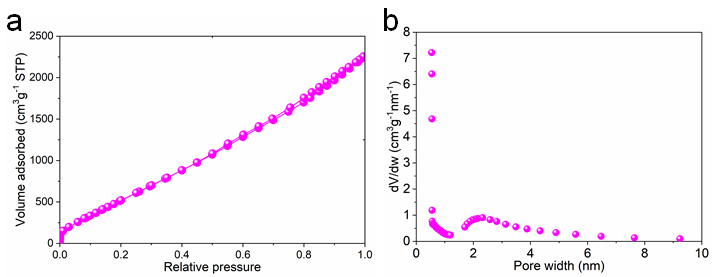


**Figure S6** (a) The isotherm curves of nitrogen adsorption/desorption and (b) pore size distribution of the HOPC carbon sample.


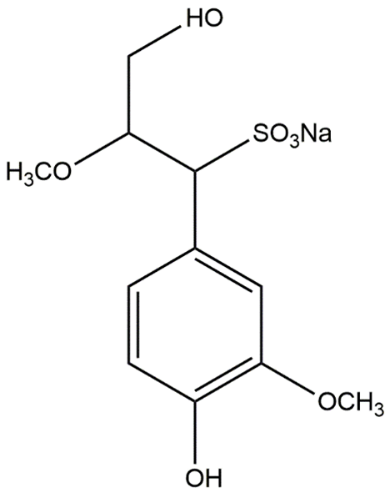


**Figure S7** Representative molecular structure of sodium lignosulphonate.


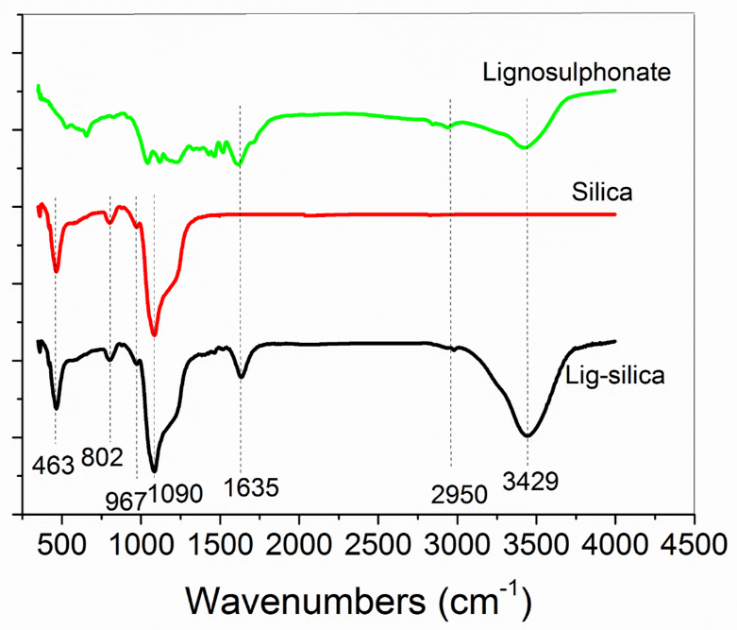


**Figure S8** The FTIR spectra of the as-prepared KIT-6 silica template, the sodium lignosulphonate and the KIT-6 template loaded with lignosulphonates (Lig-silica).


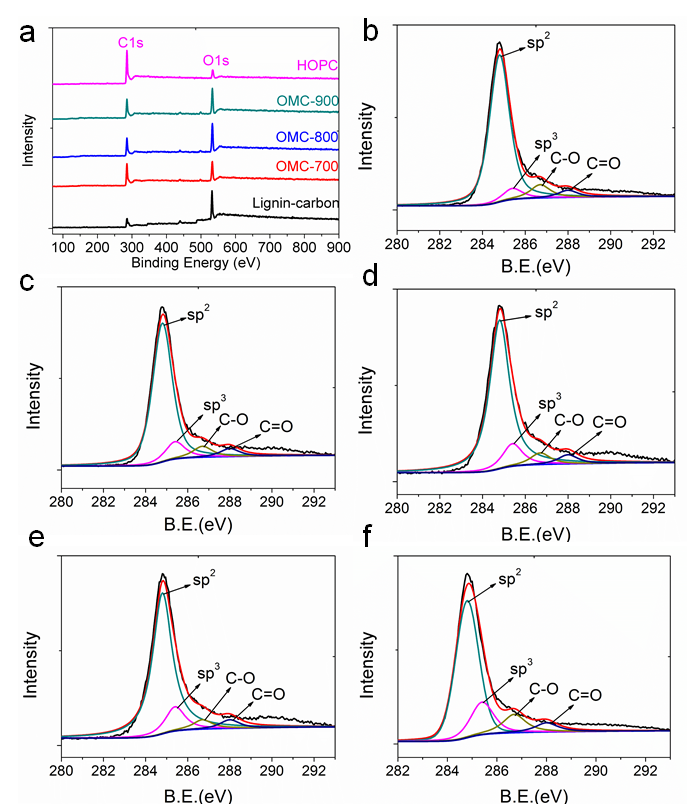


**Figure S9** XPS characterization of the as-prepared carbon samples. (a) the survey scan curves, and (b) the C1s spectra of the lignin-carbon from the carbonization of lignosulphonate without the use of template, and the C1s spectra of (c) OMC-700, (d) OMC-800, (e) OMC-900 and (d) the HOPC sample.


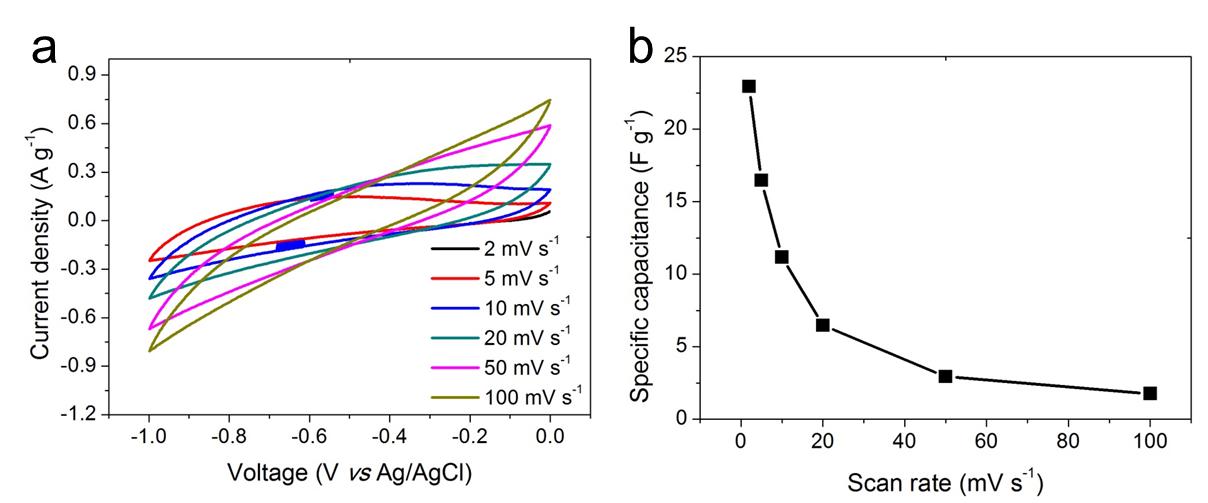


**Figure S10** CV profile and the specific capacitance of the carbon sample prepared by the direct carbonization of lignosulphonate without the use of silica template.


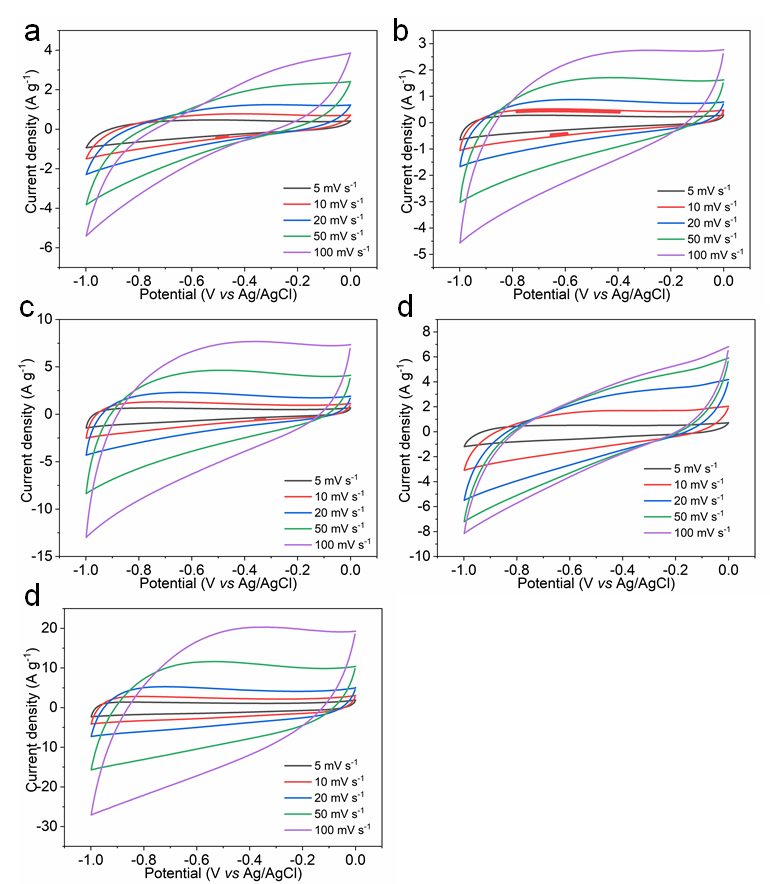


**Figure S11** CV profile of the OMC carbon samples (a) OMC-700, (b) OMC-800, (c) OMC-900, (d) OMC-1000, and the HOPC sample


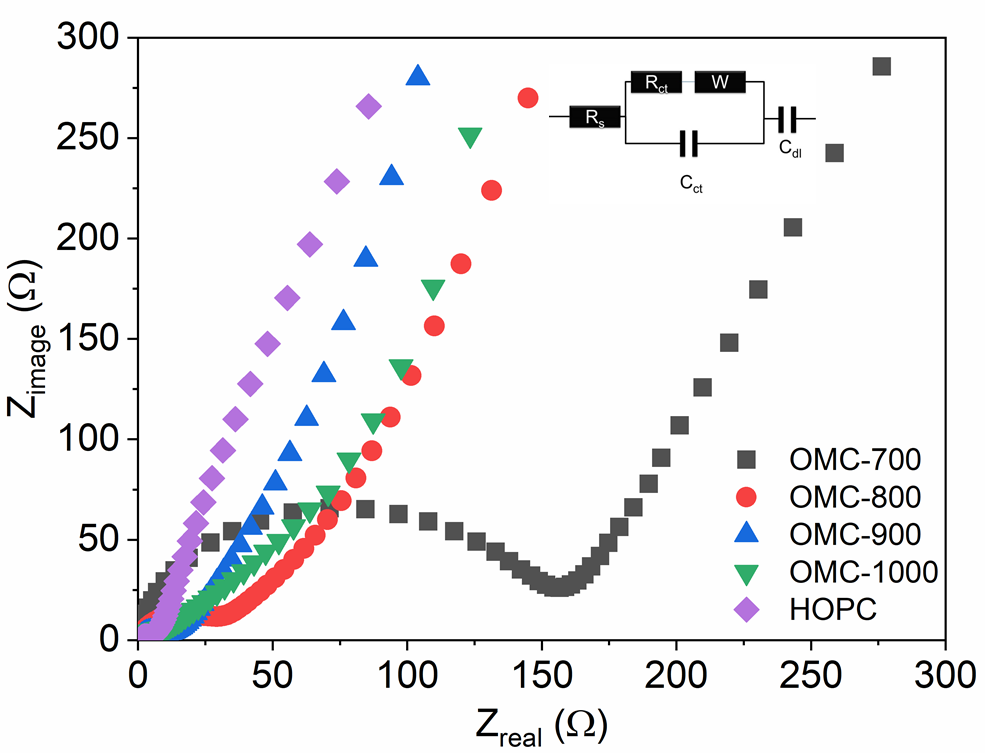


**Figure S12** The Nyquist plots of impedance in the frequency range of 1 MHz – 0.01 Hz. The insert refers to the corresponding equivalent circuit. The equivalent circuit contains an ohmic resistance R_s_, which includes the contact resistance of leads, the bulk solution resistance and the sheet resistance of the carbon film, a charge transfer capacitance C_ct_ which is in parallel with the charge transfer resistance R_ct_, and a Warburg diffusion element (W) attributed to the diffusion of electrolyte ions, and the electrical double layer capacitance C_dl_ at the low frequency region.


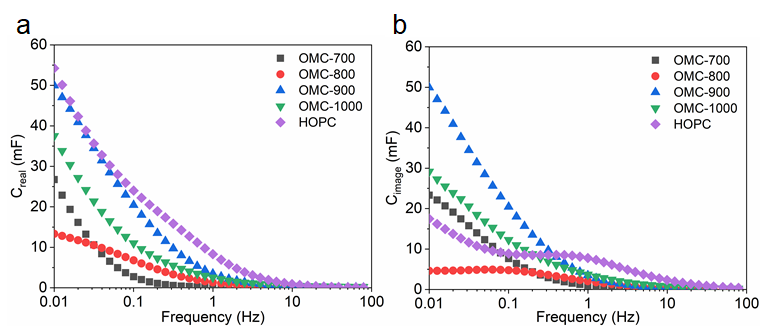


**Figure S13** (a) The real part of the capacitance C_real_ as a function of frequency and (b) the imaginary part of the capacitance C_image_ as a function of the frequency of the OMC carbon sample and the HOPC sample.


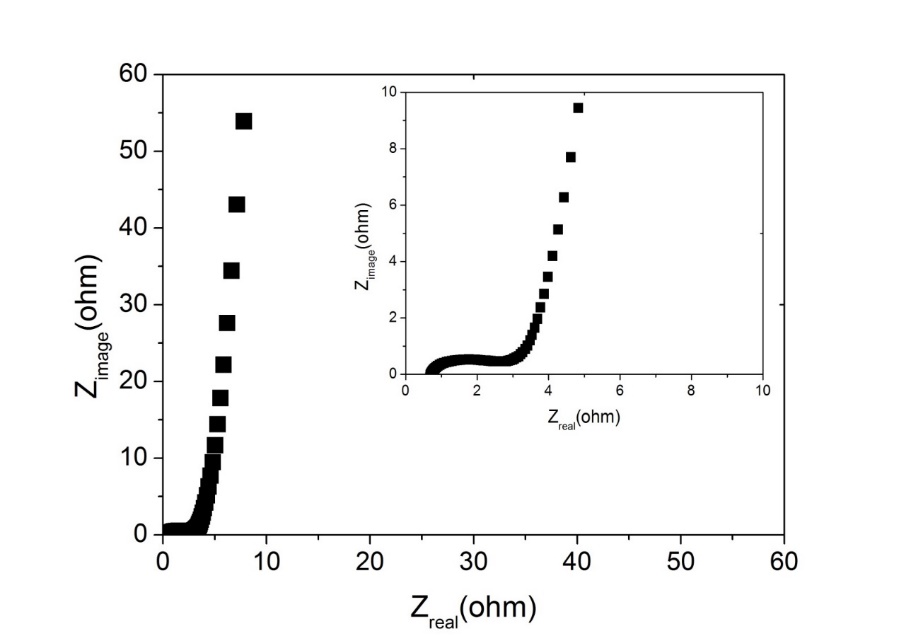


**Figure S14** Nyquist plots of the HOPC symmetric supercapacitor. The insert refers to the magnified Nyquist plots to clearly show the ohmic resistance and charge transfer resistance at high frequency and medium frequency region, respectively.


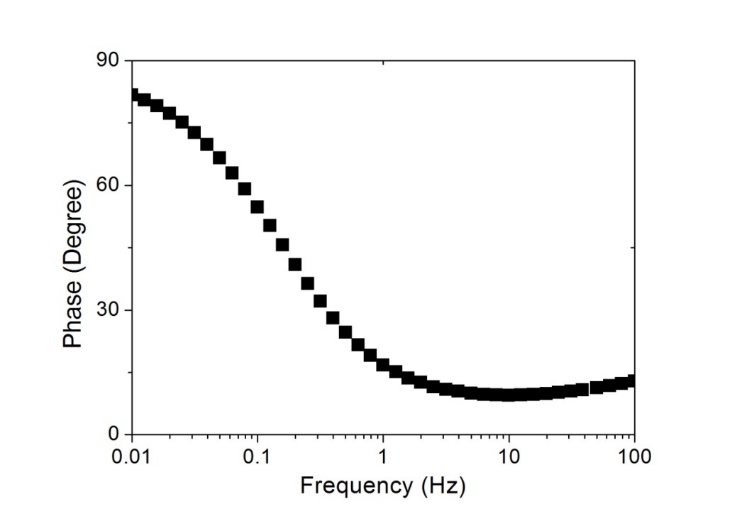


**Figure S15** The phase angle as the function of frequency of the HOPC symmetric supercapacitor.


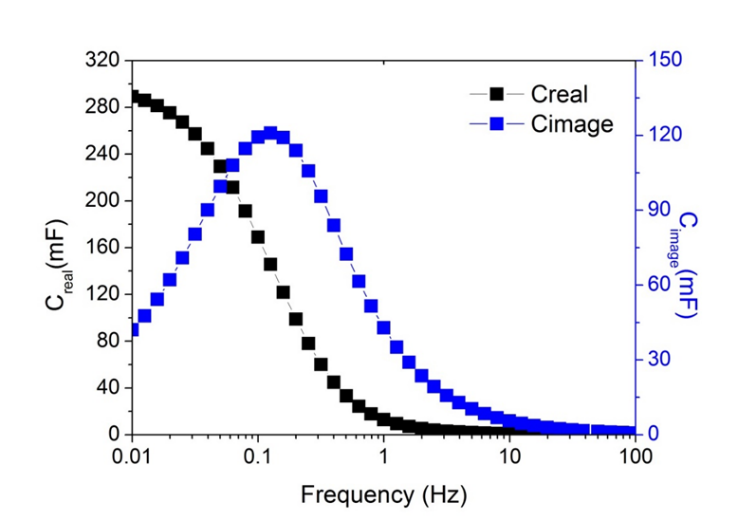


**Figure S16** C_real_ and C_image_ as the function of frequency of the HOPC symmetric supercapacitor.


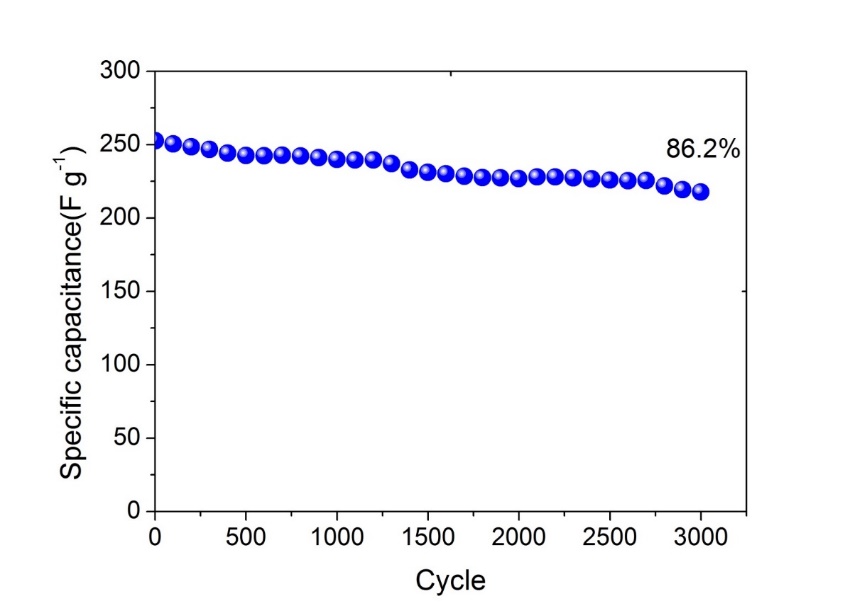


**Figure S17** Specific capacitance as a function of cycle numbers showing the capacitance retention of the HOPC symmetric supercapacitor.

**Table S2** Comparison of the specific capacitances of synthesized HOPC electroactive material with recently reported carbonaceous materials.

| **Electroactive material** | **Electrolyte** | **Test system** | **Capacitance (F g^-1^)/ Current density (A g^-1^)** | **Energy density (Wh Kg^-1^)/ Power density (W Kg^-1^)** | **Reference** |
| --- | --- | --- | --- | --- | --- |
| NCNF2-900 | 2.0 M H_2_SO_4_ | 3-electrode | 224/0.5 | 31.04/125 | [1] |
| N-CNG | 1 M H_2_SO_4_ | 3-electrode | 81/0.5 | 9.4/1542  （5.7/7462） | [2] |
| Mg8K4Ur20Cot1g-800 | 6 M KOH | 2-electrode | 279/1 | 38.7/about 1400 | [3] |
| ONS-HPCs | 6 M KOH | 2-electrode | 244.5/0.2 | 8.5/10000 | [4] |
| HSC-0.50 | 6 M KOH | 3-electrode | 281.4/0.5 | 7.6/125.1 | [5] |
| CNBs | 1 M H_2_SO_4_ | 3-electrode | 299.4/0.2 | _______ | [6] |
| HPNC-NS | ionic liquid | 2-electrode | 242/0.5 | 102/___ | [7] |
| M-NMCCs | 0.5 M K_2_SO_4_ | 2-electrode | 99.1/0.5 | 11.3/___ | [8] |
| CLCF | PVA/H_3_PO_4_ | 3-electrode | 223.8/0.5 | 5.9/1200 | [9] |
| HPCs | 6 M KOH | 2-electrode | 352/0.1 | 12.2/50 | [10] |
| hNCNC | 6 M KOH | 3-electrode | 298/1 | 10.9/6.42 | [11] |
| HMC | 1 M H_2_SO_4_ | 2-electrode | 264/0.1 | 9.1/50 | [12] |
| NODC | 6 M KOH | 2-electrode | 251/0.5 | 19.2/11160.5 | [13] |
| PF | 1 M Na_2_SO_4_ | 2-electrode | 287/0.5 | 13.9/460 | [14] |
| PC-1100 | EMIM-BF_4_ | 2-electrode | 1230/1 | 109.9/4400 | [15] |
| TRHPC | 0.5 M Na_2_SO_4_ | 2-electrode | 293/0.5 | 16/160 | [16] |
| Cu/PCNFs | 1 M H_2_SO_4_ | 2-electrode | 333.5/1 | 24.53/1500 | [17] |
| NSC | 6 M KOH | 3-electrode | 288/0.5 | 4.7/2500 | [18] |
| MoO_3_/NiCo_2_O_4_ -NSs//α-FeOOH/rGO | 3 M KOH | 2-electrode | 141/1 | 50.2/800 | [19] |
| Ni_6_MnO_8_@CNT//APDC | 1 M KOH | 3-electrode | 154/1 | 58.2/831.4 | [20] |
| **HOPC** | **6 M KOH** | **2-electrode** | **289/0.5** | **40/900** | **Present work** |

**References**

[1] Chen H., Liu T., Mou J., Zhang W., Jiang Z., Liu J., Huang J., Liu M., Free-standing N-self-doped carbon nanofiber aerogels for high-performance all-solid-state supercapacitors, Nano Energy, 63 (2019)103836.

[2] Atchudan R., Edison T.N.J.I., Perumal S., Thirukumaran P., Vinodh R., Lee Y.R., Green synthesis of nitrogen-doped carbon nanograss for supercapacitors, J Taiwan Inst Chem E, 102 (2019) 475-486.

[3] Kim C., Zhu C., Aoki Y., Habazaki H., Heteroatom-doped porous carbon with tunable pore structure and high specific surface area for high performance supercapacitors, Electrochim Acta, 314 (2019) 173-187.

[4] Liu F., Wang Z., Zhang H., Jin L., Chu X., Gu B., Huang H., Yang W., Nitrogen, oxygen and sulfur co-doped hierarchical porous carbons toward high-performance supercapacitors by direct pyrolysis of kraft lignin, Carbon, 149 (2019) 105-116.

[5] Li Z., Bai Z., Mi H., Ji C., Gao S., Pang H., Biowaste-Derived Porous Carbon with Tuned Microstructure for High-Energy Quasi-Solid-State Supercapacitors, ACS Sustain Chem Eng, 7 (2019) 13127-13135.

[6] Chang Y., Yuan C., Liu C., Mao J., Li Y., Wu H., Wu Y., Xu Y., Zeng B., Dai L., B, N co-doped carbon from cross-linking induced self-organization of boronate polymer for supercapacitor and oxygen reduction reaction, J Power Sources, 365 (2017) 354-361.

[7] Hou J., Cao C. Idrees F., Ma X., Hierarchical Porous Nitrogen-Doped Carbon Nanosheets Derived from Silk for Ultrahigh-Capacity Battery Anodes and Supercapacitors, ACS Nano, 9 (2015) 2556-2564.

[8] Liu W.J., Tian K., He Y.R., Jiang H., Yu H.Q., High-yield harvest of nanofibers/mesoporous carbon composite by pyrolysis of waste biomass and its application for high durability electrochemical energy storage, Environ Sci Technol, 48 (2014) 13951-13959.

[9] Cheng Y.L., Huang L., Xiao X., Yao G., Yuan L.Y., Li T., Hu Z.M., Wang B., Wan J., Zhou J., Flexible and cross-linked N-doped carbon nanofiber network for High performance Freestanding supercapacitor electrode, Nano Energy, 15 (2015) 66-74.

[10] Zhao G., Chen C., Yu D., Sun L., Yang C., Zhang H., Sun Y., Besenbacher F., Yu M., One-step production of O-N-S co-doped three-dimensional hierarchical porous carbons for high-performance supercapacitors, Nano Energy, 47 (2018) 547-555.

[11] Zhao J., Lai H., Lyu Z., Jiang Y., Xie K., Wang X., Wu Q., Yang L., Jin Z., Ma Y., Liu J., Hu Z., Hydrophilic Hierarchical Nitrogen-Doped Carbon Nanocages for Ultrahigh Supercapacitive Performance, Adv Mater, 27 (2015) 3541-3545.

[12] Qian W., Sun F., Xu Y., Qiu L., Liu C., Wang S., Yan F., Human hair-derived carbon flakes for electrochemical supercapacitors, Energy Environ. Sci., 7 (2014) 379-386.

[13] Gao S., Li X., Li L., Wei X., A versatile biomass derived carbon material for oxygen reduction reaction, supercapacitors and oil/water separation, Nano Energy, 33 (2017) 334-342.

[14] Liu B., Liu Y., Chen H., Yang M., Li H., Oxygen and nitrogen co-doped porous carbon nanosheets derived from Perilla frutescens for high volumetric performance supercapacitors, J Power Sources, 341 (2017) 309-317.

[15] Niu J., Shao R., Liang J., Dou M., Li Z., Huang Y., Wang F., Biomass-derived mesopore-dominant porous carbons with large specific surface area and high defect density as high performance electrode materials for Li-ion batteries and supercapacitors, Nano Energy, 36 (2017) 322-330.

[16] Wang Y., Zhao L., Peng H., Dai X., Liu X., Ma G., Lei Z., Three-dimensional honeycomb-like porous carbon derived from tamarisk roots via a green fabrication process for high-performance supercapacitors, Ionics, 25 (2019) 4315-4323.

[17] Lavanya T., Ramaprabhu S., Copper nanoparticles incorporated porous carbon nanofibers as a freestanding binder-free electrode for symmetric supercapacitor with enhanced electrochemical performance, Mater Res Express, 6 (2019) 105005.

[18] Song P., He X., Shen X., Sun Y., Li Z., Yuan A., Zhai L., Zhang D., Dissolution-assistant all-in-one synthesis of N and S dual-doped porous carbon for high-performance supercapacitors, Adv Powder Technol, 30 (2019) 2211-2217.

[19] Lin F., Yuan M., Chen Y., Huang Y., Lian J., Qiu J., Xu H., Li H., Yuan S., Zhao Y., Cao S., Advanced asymmetric supercapacitor based on molybdenum trioxide decorated nickel cobalt oxide nanosheets and three-dimensional α-FeOOH/rGO, Electrochim Acta, 320 (2019) 134580.

[20] Liu J., Xiong T., Liu T., Yang C., Jiang H., Li X., Core-shell structured Ni6MnO8@carbon nanotube hybrid as high-performance pseudocapacitive electrode material, Electrochim Acta, 320 (2019) 134627.
